# Voltammetric Detection of S100B Protein Using His-Tagged Receptor Domains for Advanced Glycation End Products (RAGE) Immobilized onto a Gold Electrode Surface

**DOI:** 10.3390/s140610650

**Published:** 2014-06-17

**Authors:** Edyta Mikuła, Aleksandra Wysłouch-Cieszyńska, Liliya Zhukova, Monika Puchalska, Peter Verwilst, Wim Dehaen, Jerzy Radecki, Hanna Radecka

**Affiliations:** 1 Institute of Animal Reproduction and Food Research of Polish Academy of Sciences, Tuwima 10, 10-748 Olsztyn, Poland; E-Mails: e.mikula@pan.olsztyn.pl (E.M.); j.radecki@pan.olsztyn.pl (J.R.); 2 Institute of Biochemistry and Biophysics of Polish Academy of Sciences, Pawińskiego 5a, 02-106 Warsaw, Poland; E-Mails: olawyslouch@ibb.waw.pl (A.W.-C.); lilia@ibb.waw.pl (L.Z.); monikapuchalska88@gmail.com (M.P.); 3 Chemistry Department, University of Leuven, Celestijnenlaan 200F, B-3001 Leuven, Belgium; E-Mails: peterverwilst@hotmail.com (P.V.); Wim.Dehaen@chem.kuleuven.be (W.D.)

**Keywords:** pentetic acid, Cu(II) complex; gold electrodes; His-tagged proteins; S100B protein; electrochemical biosensor

## Abstract

In this work we report on an electrochemical biosensor for the determination of the S100B protein. The His-tagged VC1 domains of Receptors for Advanced Glycation End (RAGE) products used as analytically active molecules were covalently immobilized on a monolayer of a thiol derivative of pentetic acid (DPTA) complex with Cu(II) deposited on a gold electrode surface. The recognition processes between the RAGE VC1 domain and the S100B protein results in changes in the redox activity of the DPTA-Cu(II) centres which were measured by Osteryoung square-wave voltammetry (OSWV). In order to verify whether the observed analytical signal originates from the recognition process between the His_6_–RAGE VC1 domains and the S100B protein, the electrode modified with the His_6_–RAGE C2 and His_6_–RAGE VC1 deleted domains which have no ability to bind S100B peptides were applied. The proposed biosensor was quite sensitive, with a detection limit of 0.52 pM recorded in the buffer solution. The presence of diluted human plasma and 10 nM Aβ_1-40_ have no influence on the biosensor performance.

## 1. Introduction

Alzheimer's disease (AD) is a progressive brain disorder characterized by memory loss and confusion [1–3]. The prevalence of AD doubles every 5 years after the age of 60 and currently affects more than 35 million patients worldwide [4,5]. The process of this disease can begin 20 years before the mind shows any signs of cognitive loss [6].

The AD brain is also characterized by elevated levels of several proteins from the S100 family, including the S100B protein [7]. It is a calcium-binding protein of the EF-hand (helix-loop-helix structure) type secreted by astrocytes and an increased concentration has been found in the serum and cerebrospinal fluid of patients with AD [8,9]. Higher levels of S100B act in the pathogenesis of neurodegenerative processes [7,10]. S100B exerts both a neurotrophic or potentially neurotoxic effect, depending of its concentration [11]. This implies that S100B overexpression significantly affects on the progression of diffuse nonfibrillar amyloid deposits to neuritic forms and consequently in the progression of the disease itself [7]. Moreover, determining serum S100B levels could be useful to distinguish the severity or observe the progression of dementia in AD [3]. Therefore, the S100B protein could be a potential biochemical marker of AD development [3,7,10,12–14].

Most, if not all, of the extracellular (neurotrophic and neurotoxic) effects observed with S100B are mediated by the Receptor for Advanced Glycation End Products (RAGE) [11,15,16]. RAGE is a multiligand receptor composed of three extracellular immunoglobulin domains: V, C1, C2, a transmembrane helix and a short (42 amino acids) tail [9] and it plays important roles in certain human pathologies such as diabetes, stroke, cancer, chronic inflammation, and neurodegenerative disorders, including AD [17–20]. Thus, in many studies RAGE has been suggested as a common extracellular receptor for S100 proteins [11,21,22]. The interaction between RAGE and the S100B protein is strictly dependent on Ca^2+^ [16]. According to the literature data the necessary concentration of Ca^2+^ enablies the binding S100B protein with RAGE receptor is range 2–20 μM [19].

The binding of Ca^2+^ causes the opening of the structure of S100B and exposes a protein-protein interaction site. The interaction site of S100B utilizes both polar and hydrophobic residues. These residues are needed for the high affinity of target proteins like RAGE [16,23]. Thus, only S100B associated with Ca^2+^ is capable to bind RAGE [16].

The conventional techniques for the determination of potential AD biomarkers are surface plasmon resonance (SPR) [8,19], enzyme-linked immunosorbent assay (ELISA) [7], and liquid chromatography [24]. However, these methods have some disadvantages. There are time-consuming, expensive, require specialized equipment and highly trained technicians. Electrochemical biosensors are analytical devices that are promising alternatives to currently used detection systems [24–31].

In this work, we describe an electrochemical biosensor based on a redox active layer consisting of a Cu(II) complex with pentetic acid (DPTA) [32]. This complex plays the role of molecular connector between the gold electrode surface and immobilized His_6_–RAGE domains as well as transducer of the intermolecular recognition process. This biosensor was applied for screening of the interactions between RAGE VC1 domain and the S100B protein. In order to verify whether the observed analytical signal originates from the recognition process between the His_6_–RAGE VC1 domains and the S100B protein, electrodes modified with the His_6_–RAGE C2 and His_6_–RAGE VC1 deletant domains which have no ability to bind S100B peptides were applied. The selectivity of the proposed biosensor was tested in the presence of a fixed concentration of Aβ_1-40_ and diluted human plasma.

## 2. Experimental Section

### 2.1. Materials and Chemicals

The thiol derivative of pentetic acid (DPTA) was synthesized at the Chemistry Department of Leuven University, Leuven, Belgium [32]. *N*-Acetylcysteamine (NAC), trizma hydrochloride (TRIS-HCl), copper (II) acetate, chloroform, potassium chloride, sodium chloride were obtained from Sigma-Aldrich (St. Louis, MO, USA). Methanol, potassium hydroxide, and sulfuric acid were purchased from POCH (Gliwice, Poland). Recombinant S100B protein was overexpressed in *E. coli* and purified as previously described [33]. Recombinant 23–223 RAGE VC1 domain with a His_6_ affinity tag attached at the proteins C-terminus was cloned in pET 28 PP plasmid (Novagen, Darmstadt, Germany) and overexpressed in BL21DE3 *E.coli* strain. Protein expression was carried out overnight at 20 °C after induction with 0.5 mM IPTG (isopropyl β-d-1-thiogalactopyranoside). Bacteria were centrifuged and the obtained pellet was lysed in 50 mM TRIS-HCl pH 8.0 with 300 mM NaCl and 10 mM imidazole. Lysozyme, phenylmethylsulfonyl fluoride (PMSF) and deoxyribonuclease (DNase) were added to improve bacterial lysis efficiency. After centrifugation the soluble fraction was used for further affinity purification on Ni-NTA columns (Qiagen, Limburg, The Netherlands). Purified protein was eluted in 50 mM TRIS-HCl pH 8.0, 300 mM imidazole and 300mM NaCl, dialyzed into TRIS-HCl buffer pH 7.4, 150 mM NaCl and purified using HiTrap Sepharose High Performance (SP HP) ion exchange column (GE Healthcare, Buckinghamshire, UK). The purity of each protein batch was confirmed using high-performance liquid chromatography (HPLC) and mass spectrometry. All aqueous solutions were prepared using MilliQ water, resistivity 18.2 MΩ cm (Millipore, Billerica, MA, USA). All solutions were deoxygenated by purging with nitrogen (ultra-pure 6.0, Warsaw, Poland) for 15 min. All experiments were carried out at room temperature.

### 2.2. Preparation of Biosensor—Successive Steps of Gold Electrode Modification

Gold disk electrodes of 2 mm^2^ area (Bioanalytical Systems (BAS), West Lafayette, IN, USA) were used for the experiments. Gold electrodes were initially cleaned mechanically by polishing with alumina slurries (Alpha and Gamma Micropolish, Buehler; Lake Bluff, IL, USA) with particles sizes of 0.3 and 0.05 μm on a micro cloth pad (BAS) for 5 min each. Afterwards, they were carefully rinsed with Milli-Q water. The polished electrodes were further cleaned electrochemically by cyclic voltammetry. At first, they were dipped in 0.5 M KOH solution and swept with a potential between −400 mV and −1200 mV against the Ag/AgCl reference electrode (with 3 M KCl inner solution) and the platinum wire counter electrode with a scan rate of 100 mV s^−1^, and the number of cycles: 3, 50 and 10, respectively. Next, the electrodes were cleaned in 0.5 M H_2_SO_4_, and cyclic voltammograms (CVs) in the potential window between −300 mV and +1500 mV were recorded, with the number of cycles: 3, 10 and 3, respectively, until the CVs underwent no further change. Before modification, the surfaces of electrodes were refreshed in a 0.5 M KOH solution for 10 cycles. Clean gold electrodes were immersed in a mixed solution of 10^−5^ M of DPTA and 10^−3^ M of NAC in an ethanol/water mixture (80:20, *v*/*v*) at 4 °C for 20 h. Next, after washing with an ethanol/water mixture, water, methanol and chloroform-methanol solution (1:1), the electrodes were dipped in 1 mM Cu (II) acetate solution in a chloroform-methanol mixture (1:1) for 3 h. Then, after washing in chloroform-methanol solution, methanol and TRIS buffer 10 μM His_6_-RAGE VC1 or C2 domains solution in TRIS buffer (10 μL) was dropped on the surface of the electrodes. The electrodes were covered and stored for 24 h at a temperature of 4 °C. The buffer composition was as follows: 300 mM TRIS–HCl, 500 mM NaCl, pH 7.4 for His_6_–RAGE VC1 natural domain; 50 mM TRIS–HCl, 300 mM NaCl, pH 7.4 for His_6_–RAGE VC1 deleted and His_6_–RAGE C2 domain. After deposition of the His_6_–RAGE VC1 or His_6_–RAGE C2 domain, the electrodes were washed and stored at 4 °C in TRIS buffer until used. Next, 10 μL of S100B solutions in TRIS buffer at concentration range: 1 pM, 5 pM, 10 pM, 15 pM, 20 pM were deposited onto the electrode surface for 30 min. After deposition of particular S100B solutions, the electrodes were carefully washed and stored in TRIS buffer until use.

### 2.3. Osteryoung Square Wave Voltammetry (OSWV) Measurements

All electrochemical measurements were performed with a potentiostat–galvanostat AutoLab (Eco Chemie, Utrecht, The Netherlands) with a conventional three electrode configuration. The following three-electrode configuration was applied: a gold (BAS) working electrode, an Ag/AgCl reference electrode and a Pt counter electrode. OSWV was performed in a potential window from +0.7 V to −0.3 V and with a step potential of 0.001 V, a square-wave frequency of 25 Hz, and amplitude of 0.05 V.

## 3. Results and Discussion

### 3.1. Characterization of Electrochemical Biosensor based on N-Acetylcysteamine/Thiol Derivative of Pentetic Acid (NAC/DPTA)–Cu(II) Monolayer

**Figure 1.**
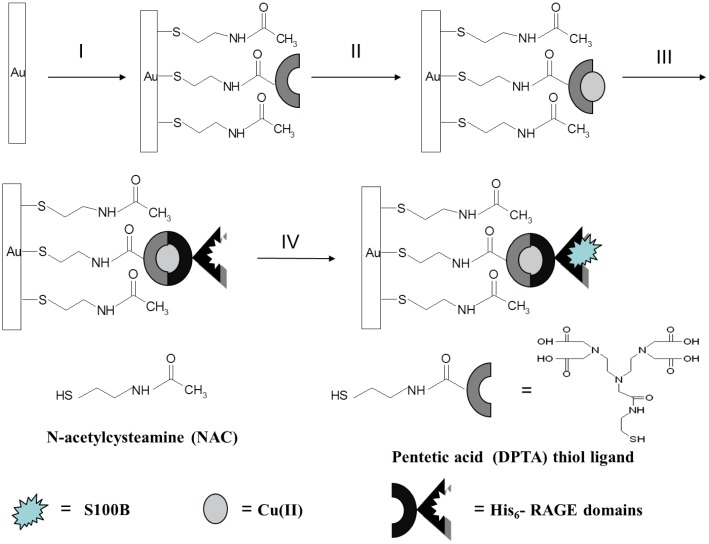
The scheme of the biosensor based on *N*-Acetylcysteamine/thiol derivative of pentetic acid (NAC/DPTA)–Cu(II) self-assembled monolayer (SAM).

Biosensors are tools that combine a biochemical binding event to a signal conversion unit and are already being used in the study of some AD biomarkers [25]. In this work, we proposed electrochemical biosensors for the determination of S100B protein. The process of the biosensor fabrication is shown in Figure 1.

First, on the Au electrode, starting from the thiol derivative of DPTA and NAC, a mixed self assembled monolayer was formed. Then, Cu(II) was complexed by DPTA attached to the surface of the Au electrode. His_6_–RAGE VC1 domain was covalently attached via coordination bonds between Cu(II) sites from DPTA–Cu(II) complex and imidazole nitrogen atoms of a histidine tag [32]. In order to provide the best accessibility of DPTA for Cu(II) ions and gain proper isolation of redox centres from one another, a mixed monolayer was applied. The thiol derivative of DPTA was diluted with NAC, which serves as spacer molecules to avoid intramolecular interaction between the Cu(II) redox centers. Thanks to the peptide bonds, NAC molecules form a hydrogen bonded network, which makes the monolayer more stable and better organized [34].

**Figure 2.**
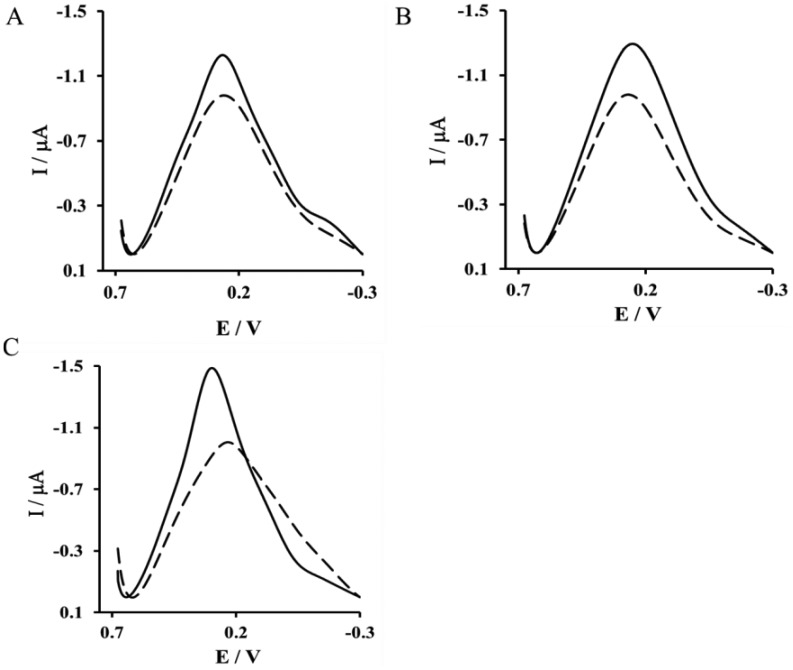
Osteryoung square wave voltammograms of a gold electrode modified by NAC/DPTA–Cu(II) (solid line) and after immobilization of: (**A**) RAGE VC1 natural domain; (**B**) RAGE VC1 deleted domain; and (**C**) RAGE C2 domain (dashed lines). Measurement conditions: 0.1 M KCl, scan rate100 mV·s^−1^.

The immobilization of His_6_–RAGE VC1 (natural and deleted) or His_6_–RAGE C2 domains on the surface of a NAC/DPTA–Cu(II) monolayer were monitored by Osteryoung square-wave voltammetry (OSWV). In this technique the current is measured at the end of each potential change, right before the next, so that the contribution to the current signal from the capacitive charging current is minimized [35]. The Cu(II) redox peak current was observed at 298 mV ± 17 mV. The complexation reaction between Cu(II) and His_6_–RAGE domains caused a decrease of the Cu(II) redox peak (Figure 2A–C).

### 3.2. Exploring the Interactions between the VC1and C2 RAGE Domains and the S100B Peptides Using OSWV

**Figure 3.**
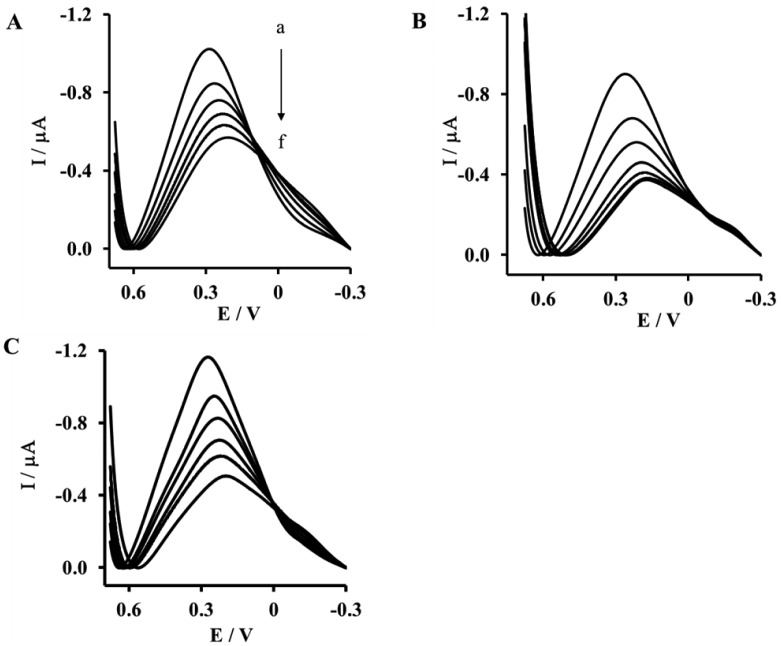
The representative Osteryoung Square Wave Voltammetry (OSWV) responses measured with electrodes: (**A**) Au-NAC/DPTA–Cu(II)–His_6_–RAGE VC1 natural domain in TRIS buffer in the presence of Ca^2+^; (**B**) Au-NAC/DPTA–Cu(II)–His_6_–RAGE VC1 natural domain in diluted human plasma in the presence of Ca^2+^ and (**C**) Au-NAC/ DPTA–Cu(II)–His_6_–RAGE VC1 natural domain in TRIS buffer with 10 nM Aβ_1-40_ in the presence of Ca^2+^ towards S100B protein in the presence of 50 mM TRIS-HCl, pH 7.4. (**a**) 0.0; (**b**) 1.0; (**c**) 5.0; (**d**) 10.0; (**e**) 15.0; and (**f**) 20.0 [pM].

The presented biosensor was applied for screening of interactions between the His_6_- RAGE VC1 domain and the S100B protein. The mechanism of signal generation is similar to the one presented in a previous paper concerning the biosensor destined for determination of Aβ_1-40_ and Aβ_16-23_ [32]. According to this, a RAGE VC1 domain conformation change and charge redistribution, which occurred upon binding of the S100B protein, caused a restriction of the counter ions accessing the redox centres to balance the charge, thereby hindering the electron transfer. As the consequence, upon binding of the S100B protein, a decrease of the Cu(II) redox current was observed (Figure 3A–C, Table 1). The idea of charge redistribution responsible for signal transduction has been recently reported by Stobiecka and Hepel [36].

Upon increasing of protein S100B concentration we have observed the decreasing of Cu(II)/Cu(III) redox current assisted with potential shift into the negative direction. This phenomenon is in a good agreement with the theoretical elucidation of the mechanism of a surface electrode reaction mechanism under conditions of square—wave voltammetry presented by Gulaboski *et al.* [37].

The difference in peak current recorded in the buffer solution is caused by the different amount of Cu(II) centres attached to the electrode surface. Therefore, in order to eliminate the differences in electrode modification we have used the relative values of Cu(II) current decrease: [(*I_i_*–*I*_0_)/*I*_0_] × 100%, where: *I*_0_ is the Cu(II) redox current measured in the presence of buffer; *I_i_* is the Cu(II) redox current measured in the presence of a particular concentration of S100B protein.

The highest concentration of the S100B protein caused a 61.7% ± 5.2% decrease of the peak current recorded for the electrode incorporating the His_6_–RAGE VC1 natural domain measured in TRIS buffer in the presence of Ca^2+^ (Figure 3A, Table 1). Similar result was observed for His_6_–RAGE VC1 natural domain recorded in the presence of diluted human plasma (56.1% ± 7.2%) (Figure 3B, Table 1) and in the presence of 10 nM Aβ_1-40_ (55.5% ± 4.7%) (Figure 3C, Table 1). These results confirmed the biosensor selectivity. The human plasma components, as well as Aβ_1-40_ have no influence of S100B protein determination. This is very important from the diagnostic point of view.

**Figure 4.**
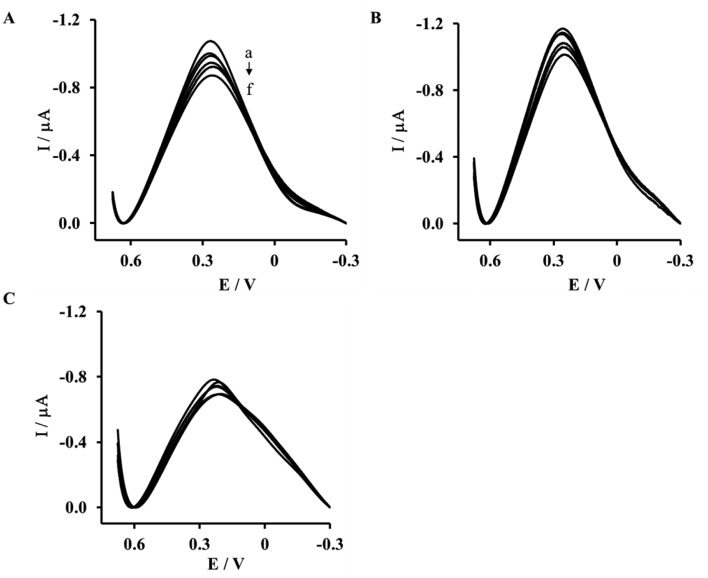
The representative Osteryoung Square Wave Voltammetry (OSWV) responses measured with electrodes: (**A**) Au-NAC/DPTA–Cu(II)–His_6_–RAGE VC1 deleted domain in TRIS buffer in the presence of Ca^2+^; (**B**) Au-NAC/DPTA–Cu(II)–His_6_–RAGE VC1 natural domain in TRIS buffer without Ca^2+^ and (**C**) Au-NAC/DPTA–Cu(II)–His_6_–RAGE C2 domain in TRIS buffer in the presence of Ca^2+^ towards S100B protein: (**a**) 0.0; (**b**) 1.0; (**c**) 5.0; (**d**) 10.0; (**e**) 15.0; and (**f**) 20.0 [pM]. Buffer composition: 50 mM TRIS-HCl, pH 7.4.

In order to check the biosensor specificity, we performed three control experiments with the His_6_–RAGE VC1 natural domain in TRIS buffer without Ca^2+^, His_6_–RAGE C2 and His_6_–RAGE VC1 deleted domain (in TRIS buffer with Ca^2+^) (Figure 4A–C, Table 1). In these conditions, the S100B protein should not be recognized and no analytical signal should be generated.

The electrode incorporating the His_6_–RAGE C2 domain responded only a little towards S100B protein (Figure 4C), with maximum current decrease 11.5% ± 1.5% (Table 1). A similar current decrease was recorded in buffer solution free of Ca^2+^ ions for an electrode modified with His_6_–RAGE VC1 (Figure 4B, Table 1).

For an electrode incorporating the His_6_–RAGE VC1 deleted domain, a higher signal was recorded (Figure 4A, Table 1). This might be caused by incomplete removal of S100B protein-binding sites. These data proved the specificity of the biosensor presented. The signals generated in the presence of S100B with the electrode incorporating the His_6_–RAGE VC1 natural domain recorded in TRIS buffer without Ca^2+^ were five times lower in the comparison to those recorded in the presence of Ca^2+^ (Figure 4B, Table 1). These results confirmed that the presence of Ca^2+^ is necessary to make the interaction between RAGE VC1 and S100B possible.

**Figure 5.**
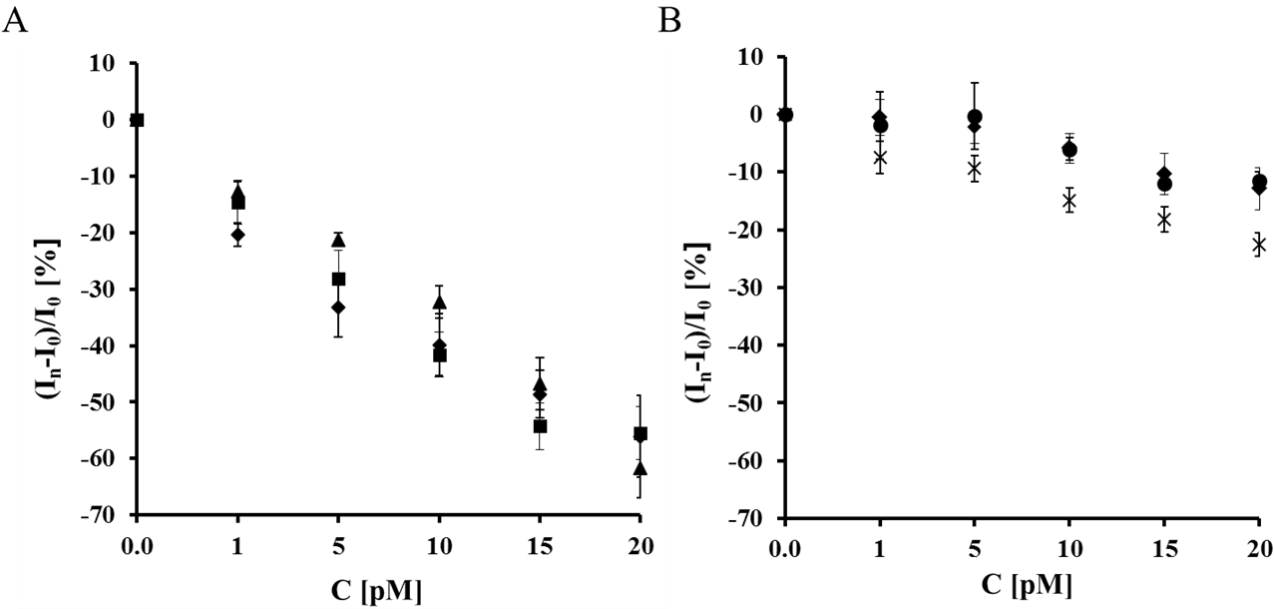
The relationship of relative Cu(II) redox current decrease *vs.* concentration of S100B measured with electrodes: (**A**) (▲) Au/NAC/DPTA–Cu(II)–His_6_–RAGE VC1 natural domain in TRIS buffer in the presence of Ca^2+^; (♦) Au/NAC/DPTA–Cu(II)–His_6_–RAGE VC1 natural domain in diluted human plasma in the presence of Ca^2+^; (■) Au/NAC/IDA–Cu(II)–His_6_–RAGE VC1 natural domain in TRIS buffer with 10 nM Aβ_1-40_ in the presence of Ca^2+^. (**B**) (●) Au/NAC/DPTA–Cu(II)–His_6_–RAGE C2 domain in TRIS buffer in the presence of Ca^2+^; (×) Au/NAC/DPTA–Cu(II)–His_6_–RAGE VC1 deleted domain in TRIS buffer in the presence of Ca^2+^; (♦) Au/NAC/IDA–Cu(II)–His_6_–RAGE VC1 natural domain in TRIS buffer without Ca^2+^.

**Figure 6.**
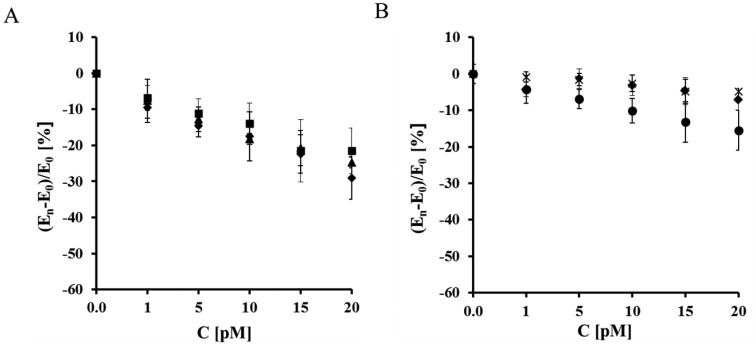
The relationship of (*E*_n_ − *E*_o_)/*E*_o_ (%) *vs.* concentration of S100B measured with electrodes: (**A**) (▲) Au/NAC/DPTA–Cu(II)–His_6_–RAGE VC1 natural domain in TRIS buffer in the presence of Ca^2+^; (♦) Au/NAC/DPTA–Cu(II)–His_6_–RAGE VC1 natural domain in diluted human plasma in the presence of Ca^2+^; (■) Au/NAC/IDA–Cu(II)–His_6_–RAGE VC1 natural domain in TRIS buffer with 10 nM Aβ_1-40_ in the presence of Ca^2+^. (**B**) (●) Au/NAC/DPTA–Cu(II)–His_6_–RAGE C2 domain in TRIS buffer in the presence of Ca^2+^; (×) Au/NAC/DPTA–Cu(II)–His_6_–RAGE VC1 deleted domain in TRIS buffer in the presence of Ca^2+^; (♦) Au/NAC/IDA–Cu(II)–His_6_–RAGE VC1 natural domain in TRIS buffer without of Ca^2+^.

The sensitivity of the analytical system based on a gold electrode modified with NAC/ DPTA–Cu(II)-His_6_-RAGE VC1 domain towards S100B protein was very good. The relative Cu(II) redox current decreased, as well as the potential shift correlated well with the S100B concentration as a linear function (Figures 5 and 6).

A decrease of the Cu(II) redox peak current assisted by a peak potential shift into the negative potential direction was observed in the whole S100B concentration range (Figures 5A and 6A). The presence of 10 nM Aβ_1-40_ and diluted human plasma did not have an influence on the S100B determination. On the other hand in the case of all control experiments incorporating the His_6_–RAGE VC1 natural domain in TRIS buffer without Ca^2+^, His_6_–RAGE C2 domain and His_6_–RAGE VC1 deleted domain, the decrease of Cu(II) peak current was negligible (Figure 5B, Table 1) and the peak potential remained almost unchanged in the presence of S100B in the whole concentration range (Figure 6B, Table 2).

**Figure 7.**
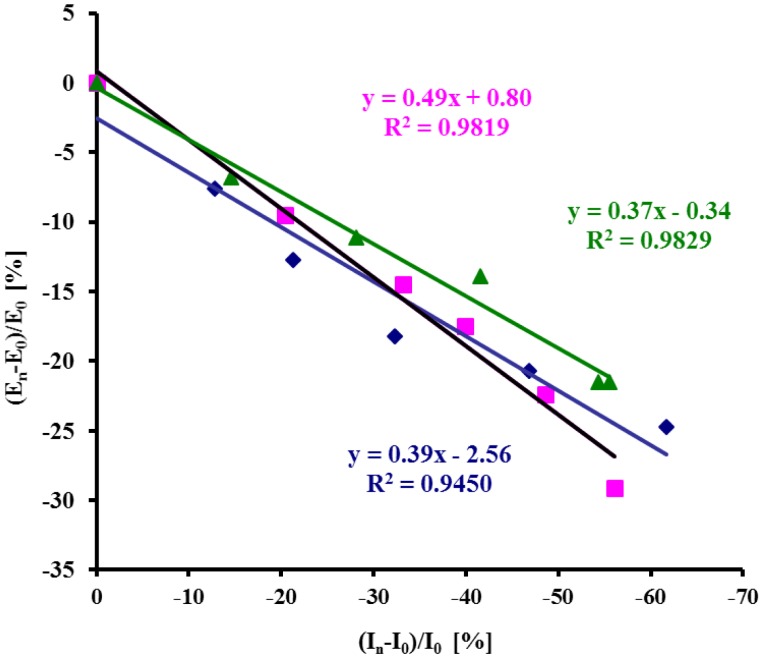
The relationship of the relative Cu(II) redox current decrease and the peak potential shift (%) *vs.* the concentration of S100B measured with electrodes: (♦) Au/NAC/DPTA–Cu(II)–His_6_–RAGE VC1 natural domain in TRIS buffer in the presence of Ca^2+^; (■) Au/NAC/DPTA–Cu(II)–His_6_–RAGE VC1 natural domain in diluted human plasma in the presence of Ca^2+^; (▲) Au/NAC/DPTA–Cu(II)–His_6_–RAGE VC1 natural domain in TRIS buffer with 10 nM Aβ_1-40_ in the presence of Ca^2+^.

In general, the half peak width of the voltammograms increased with the increasing concentration of protein S100B indicating the decreasing of reversibility (Table 3). Both the parameters: relative Cu(II) redox current decrease and Cu(II) peak potential shift correlated very well (Figure 7). Thus, both of them could be applied for the determination of S100B protein in physiological samples. This substantially improves the reliability of samples analysis.

The detection limits (DL) were calculated on the basis of equation (*S*/*N* = 3.0) [38]:

(1) DL=3.3σ/s

where: σ is the standard deviation of the response and s is the slope of the calibration curve.

The obtained values were 0.52 pM, 1.1 pM and 0.65 pM, in the presence in buffer, 10 nM Aβ_1-40_ and diluted human plasma, respectively. The concentration of S100B relevant from the medical point of view is in the range 8.4–16.0 pM [7]. Thus, the detection limits calculated for biosensor presented indicated its suitability for determination of S100B protein in physiological samples.

## 4. Conclusions/Outlook

The electrochemical biosensor incorporating NAC/DPTA-Cu(II)-His_6_–RAGE VC1 domain was suitable for the determination of S100B protein. A decrease of the redox Cu(II) current, assisted with a peak potential shift were observed in the whole concentration range from 1 pM to 20 pM. A good correlation between these two analytical parameters might improve substantially the biosensor reliability. The presence of human plasma and another potential marker for early diagnosis of Alzheimer's disease (Aβ_1-40_) have no influence on the biosensor performance. The detection limit was in the pM range. Taking into account the above parameters, the proposed biosensor could be become one of the analytical tools suitable for medical samples analysis.

## Figures and Tables

**Table 1.** The relative Cu(II) redox changes (%) current measured with electrodes incorporating His_6_–RAGE domains (VC1, VC1 deleted and C2) after incubation with S100B solution. (*I*_n_ is the current measured in the presence on a particular S100B concentration, *I*_0_ is the current measured in buffer with no analyte).

	**S100B Protein in TRIS Buffer**	**S100B Protein in TRIS Buffer with Aβ_1-40_**	**S100B Protein in the Presence of Diluted Human Plasma**	**S100B Protein in TRIS Buffer**		
in the presence of Ca^2+^						without Ca^2+^

	His_6_–RAGE VC1 natural	His_6_–RAGE VC1 natural	His_6_–RAGE VC1 natural	His_6_–RAGE C2	His_6_–RAGE VC1 deleted	His_6_–RAGE VC1 natural

C (pM)	(*I*_n_ – *I*_0_)/*I*_0_ [%]	(*I*_n_ – *I*_0_)/*I*_0_ [%]	(*I*_n_ – *I*_0_)/*I*_0_ [%]	(*I*_n_ – *I*_0_)/*I*_0_ [%]	(*I*_n_ – *I*_0_)/*I*_0_ [%]	(*I*_n_ – *I*_0_)/*I*_0_ [%]
0	0	0	0	0	0	0
1	−12.8 ± 1.9	−14.6 ± 3.9	−20.4 ± 2.1	−1.9 ± 5.8	−7.5 ± 2.8	−0.6 ± 3.1
5	−21.3 ± 1.2	−28.1 ± 4.9	−33.2 ± 5.3	−0.3 ± 5.7	−9.4 ± 2.3	−2.3 ± 2.7
10	−32.3 ± 2.8	−41.6 ± 4.0	−39.9 ± 5.5	−6.0 ± 2.0	−14.9 ± 2.1	−5.9 ± 2.5
15	−46.8 ± 4.6	−54.3 ± 4.1	−48.6 ± 4.2	−12.0 ± 0.9	−18.2 ± 2.2	−10.3 ± 3.6
20	−61.7 ± 5.2	−55.5 ± 4.7	−56.1 ± 7.2	−11.5 ± 1.5	−22.6 ± 2.0	−12.9 ± 3.7

**Table 2.** The Cu(II) peak potential shift (%) measured with electrodes incorporating His_6_–RAGE domains (VC1, VC1 deleted and C2) after incubation with S100B solution. *E*_n_ is the peak potential measured in the presence of particular peptide concentration, *E*_0_ is the peak potential measured in buffer with no analyte.

	**S100B Protein in TRIS Buffer**	**S100B Protein in TRIS Buffer with Aβ_1-40_**	**S100B Protein in the Presence of Diluted Human Plasma**	**S100B Protein in TRIS Buffer**		
in the presence of Ca^2+^						without Ca^2+^

	His_6_–RAGE VC1 natural	His_6_–RAGE VC1 natural	His_6_–RAGE VC1 natural	His_6_–RAGE C2	His_6_–RAGE VC1 deleted	His_6_–RAGE VC1 natural

C (pM)	(*E*_n_ – *E*_0_)/*E*_0_ [%]	(*E*_n_ – *E*_0_)/*E*_0_ [%]	(*E*_n_ – *E*_0_)/*E*_0_ [%]	(*E*_n_ – *E*_0_)/*E*_0_ [%]	(*E*_n_ – *E*_0_)/*E*_0_ [%]	(*E*_n_ – *E*_0_)/*E*_0_ [%]
0	0	0	0	0	0	0
1	−7.6 ± 6.0	−6.8 ± 3.5	−9.5 ± 2.9	−4.3 ± 3.8	−0.9 ± 2.6	−4.3 ± 2.0
5	−12.7 ± 3.4	−11.1 ± 4.1	−14.5 ± 3.2	−6.9 ± 2.6	−1.6 ± 1.4	−1.3 ± 2.7
10	−18.2 ± 1.5	−13.9 ± 5.7	−17.5 ± 6.8	−10.1 ± 3.4	−2.6 ± 1.7	−3.2 ± 2.8
15	−20.7 ± 4.9	−21.5 ± 8.7	−22.4 ± 5.3	−13.2 ± 5.5	−4.8 ± 2.2	−4.7 ± 3.7
20	−24.7 ± 1.6	−21.5 ± 6.3	−29.1 ± 5.9	−15.5 ± 5.5	−4.9 ± 3.3	−7.1 ± 3.0

**Table 3.** The half peak width Δ*E*_1/2_ (mV) measured with electrodes incorporated His_6_–RAGE C2 and VC1 domains after incubation with S100B solution.

	**S100B Protein in TRIS Buffer**	**S100B Protein in TRIS Buffer with Aβ_1-40_**	**S100B Protein in the Presence of Diluted Human Plasma**	**S100B Protein in TRIS Buffer**		
in the presence of Ca^2+^						without Ca^2+^

	His_6_–RAGE VC1 natural	His_6_–RAGE VC1 natural	His_6_–RAGE VC1 natural	His_6_–RAGE C2	His_6_–RAGE VC1 deleted	His_6_–RAGE VC1 natural

C (pM)	Δ*E*_1/2_	Δ*E*_1/2_	Δ*E*_1/2_	Δ*E*_1/2_	Δ*E*_1/2_	Δ*E*_1/2_
0	391.0 ± 10.4	389.5 ± 3.3	394.8 ± 23.0	348.0 ± 9.8	368.3 ± 3.1	385.0 ± 14.0
1	409.8 ± 6.7	395.0 ± 6.3	418.3 ± 7.5	337.0 ± 20.2	378.3 ± 1.2	391.0 ± 11.5
5	427.8 ± 11.0	409.8 ± 5.9	415.8 ± 24.9	328.7 ± 28.5	383.0 ± 1.0	397.0 ± 9.2
10	452.0 ± 6.5	430.3 ± 5.9	411.4 ± 17.4	350.3 ± 9.3	392.0 ± 3.0	399.3 ± 6.8
15	471.8 ± 9.7	460.8 ± 5.3	420.7 ± 13.6	378.0 ± 18.3	398.0 ± 2.6	405.3 ± 3.1
20	487.3 ± 10.6	478.7 ± 8.1	426.7 ± 10.8	391.7 ± 7.1	408.3 ± 6.4	409.3 ± 3.1
